# Exosomal miR-223-3p from bone marrow mesenchymal stem cells targets HDAC2 to downregulate STAT3 phosphorylation to alleviate HBx-induced ferroptosis in podocytes

**DOI:** 10.3389/fphar.2024.1327149

**Published:** 2024-02-20

**Authors:** Yueqi Chen, Xiaoqian Yang, Moxuan Feng, Yani Yu, Yongzheng Hu, Wei Jiang

**Affiliations:** Department of Nephrology, The Affiliated Hospital of Qingdao University, Qingdao, Shandong, China

**Keywords:** ferroptosis, hepatitis B virus associated glomerulonephritis, bone marrow mesenchymal stem cells, exosome, MiR-223-3p

## Abstract

**Background:** Hepatitis B virus associated-glomerulonephritis (HBV-GN) is one of the major secondary renal diseases in China, and microRNAs (miRNAs) in bone marrow mesenchymal stem cell-derived exosomes (BMSC-Exo) can attenuate HBV-X protein (HBx)-induced ferroptosis in renal podocytes, but the exact mechanism remains unclear. This study aimed to investigate the protective mechanism of miR-223-3p in BMSC-Exo in HBx-induced ferroptosis in podocytes.

**Methods:** The study employed human renal podocyte cells (HPCs), bone marrow-derived mesenchymal stem cells (BMSCs), as well as kidney tissue from C57BL/6 mice and HBx transgenic mice. Initially, the correlation between STAT3 phosphorylation and ferroptosis was authenticated through the administration of signal transducer and activator of transcription 3 (STAT3) phosphorylation inhibitors in both *in vivo* and *in vitro* settings. Furthermore, the effect of HDAC2 overexpression on STAT3 phosphorylation was examined. Subsequently, the association between BMSC-Exo carrying miR-223-3p, HDAC2, and the phosphorylation of STAT3 in HPCs ferroptosis and injury induced by HBx was assessed. The interaction between miR-223-3p and HDAC2 was confirmed via RNA immunoprecipitation assay. Various techniques such as cell counting kit-8 assay, western blot, RT-qPCR, immunofluorescence, flow cytometry, lipid peroxidation assay kit, iron assay kit, transmission electron microscopy, and hematoxylin-eosin staining were employed to visualize the extent of HBx-induced podocyte injury and ferroptosis in both *in vivo* and *in vitro*.

**Results:** The attenuation of podocyte ferroptosis can be achieved by inhibiting the phosphorylation of STAT3 in podocytes induced by HBx. Conversely, the upregulation of HDAC2 can enhance STAT3 phosphorylation, thereby promoting podocyte ferroptosis. MiR-223-3p was capable of directly exerting negative regulation on HDAC2 expression. BMSC-Exo carrying miR-223-3p can effectively suppress the expression of HDAC2, ultimately leading to reduce HBx-induced ferroptosis in podocytes by targeting HDAC2 with miR-223-3p and downregulating STAT3 phosphorylation.

**Conclusion:** This study evidences the potential of BMSC-Exo mediated delivery of miR-223-3p in mitigating HBx-induced ferroptosis in podocytes, thereby offering a novel therapeutic target and approach for treating HBV-GN and alleviating renal injury.

## 1 Introduction

China is a high-prevalence area of hepatitis B virus (HBV), and HBV infection may give rise to a variety of extrahepatic diseases, such as HBV-associated glomerulonephritis (HBV-GN). The indispensable role of HBV-X protein (HBx) in HBV-GN has been established (Cacoub and Asselah, 2022). HBV-GN frequently manifests with glomerular injury, encompassing various pathological types such as membranous nephropathy, membranoproliferative glomerulonephritis, and mesangial proliferative glomerulonephritis (Liu et al., 2015). Podocytes, integral components of the glomerular filtration barrier, represent a significant focal point in the pathogenesis of HBV-GN; however, the precise mechanism underlying podocyte injury remains unclear.

Mesenchymal stem cells (MSCs) have become the most commonly employed cell type in cell therapy, and present in various tissues such as bone marrow, umbilical cord, and adipose tissue (Galipeau and Sensébé, 2018). The paracrine activity of MSCs partly occurs through the release of extracellular vesicles (EVs), including exosomes and microvesicles (Keshtkar et al., 2018). Exosomes, which are subcellular vesicles ranging from 40 to 160 nm in diameter and encapsulated by lipid bilayers, can be found in nearly all body fluids (Kalluri and LeBleu, 2020). Exosomes can transport and deliver signaling molecules that control the physiological states of cells and are closely associated with the progression and development of acute kidney injury (Zhang et al., 2021) and chronic kidney disease (Yun and Lee, 2019) via modulation of inflammation, oxidative stress, fibrosis, and apoptosis. Notably, EVs consisting of microRNAs (miRNAs) secreted by MSCs exhibit a protective effect on renal function in chronic kidney disease (Wang et al., 2020). Furthermore, bone marrow mesenchymal stem cells (BMSCs) can mitigate acute kidney injury caused by ischemia-reperfusion through miR-223 (Yuan et al., 2017). Furthermore, our previous investigations unveiled that restraint of miR-223 exhibited a propensity to enhance HBx-induced podocyte pyroptosis via the NLRP3 inflammasome, indicating the potential significance of miR-223 in HBx-induced podocytes (Yu et al., 2022).

Ferroptosis, characterized by accumulation of iron-dependent lipid peroxidation to lethal levels, is a form of regulated cell death that contributes to the pathogenesis of various diseases such as acute liver failure due to HBV infection and acute kidney injury due to ischemia-reperfusion (Dixon et al., 2012; Tonnus et al., 2021; Liu et al., 2021). Notably, HBx is closely linked to oxidative stress and lipid peroxidation, which are integral to the process of ferroptosis (Yi et al., 2003). Comparison of the differences in protein expression profiles between the kidneys of HBV transgenic mice and the corresponding wild mice revealed that downregulation of the solute carrier (SLC) family was closely associated with HBV expression (Zuo et al., 2020). SLC7A11, the 11th member of solute transporter family 7, belongs to the cystine/glutamate retrograde transporter proteins, and is also a key regulator of ferroptosis. We speculate that ferroptosis may be critically involved in HBV-GN kidney injury.

Initially, it was discovered that miR-223 regulates the expression and activity of HDAC2 in lung cells affected by COPD (Leuenberger et al., 2016). Additionally, studies on the immunopathogenesis of COVID-19 revealed that miR-223 reduces the expression of HDAC2 (Houshmandfar et al., 2021). A recent investigation demonstrated that miR-223-3p mitigates inflammation and cellular pyroptosis induced by AKI by targeting HDAC2 and promoting SNRK transcription. Notably, miR-223-3p is derived from BMSCs-derived exosome (BMSC-Exo) (Xie et al., 2023). miR-223 is highly expressed in BMSC and BMSC-Exo protects against liver injury in a mouse model of autoimmune hepatitis (Chen et al., 2018). We further explored the protective role of miR-223-3p in BMSC-Exo, which may provide a new target of action for the treatment of HBV-GN. Our preliminary research has indicated a potential association between ferroptosis and HBx-induced podocyte injury, and we have identified miR-223-3p in BMSC-Exo as a possible therapeutic agent to mitigate this damage (Yang et al., 2023). Previous studies have demonstrated the significant role played by STAT3 phosphorylation in ferroptosis, which has been implicated in the pathogenesis of numerous diseases (Luo et al., 2023). However, the connection between STAT3 phosphorylation and ferroptosis in podocytes has not yet been investigated. Therefore, in order to gain a deeper understanding of how miR-223-3p in BMSC-Exo may suppress ferroptosis and alleviate injury in renal podocytes, we aim to identify downstream targets of miR-223-3p and explore the relationship between STAT3 phosphorylation and ferroptosis in podocytes. We also seek to determine whether this relationship plays a role in the therapeutic benefits of BMSC-Exo.

## 2 Materials and methods

### 2.1 Purification, characterization, transfection, and uptake of BMSC-Exo

Mouse BMSCs (CP-M131) was acquired from Procell (Wuhan) and cultured in RPMI-1640 medium (Procell, PM150110, Wuhan) supplemented with 10% fetal bovine serum and 100 U/mL penicillin/streptomycin (meilunbio, Dalian). Mouse BMSCs were seeded into 24-well plates at a density of 1.2×10^5^ cells per well and transfected with Lipofectamine^®^3000 reagent (Thermo Fisher Scientific Waltham, MA, United States) following the provided instructions. BMSCs were transfected with histone deacetylase 2 (HDAC2) overexpressed and empty plasmid, and miR-223-3p inhibitor.

To obtain BMSC-Exo, mouse BMSCs was cultured in serum-free RPMI-1640 medium supplemented with 100 U/mL penicillin/streptomycin, and the supernatant was collected 48 h later. The supernatant was subjected to low-speed centrifugation (2,000 × g, 30 min; 10,000 × g, 45 min, 4°C) and filtered using a 0.45 μm filter membrane (Millipore, R6BA09493). The filtrate underwent dual rounds of ultracentrifugation at 100,000 × g and 4°C for 70 min per cycle utilizing a centrifuge (Hitachi, CP100MX). The purified BMSC-Exo was resuspended in 300 μL PBS, of which 20 μL was utilized for observing the morphology of BMSC-Exo utilizing the transmission electron microscopy (TEM), 10 μL was used for measuring the particle size and concentration of BMSC-Exo using NanoFCM (N30E, Fujian), and 40 μL was used for total protein extraction. The expressions of exosome markers (TSG101, CD9) were examined by western blot analysis. Additionally, 100 μL was taken to assess the uptake of BMSC-Exo by podocytes, and the red dye PKH26 labeled BMSC-Exo (PBS as the control) was co-cultured with podocytes, followed by staining with 4′,6-diaminyl-2-phenylindole (Dapi). Imaging was performed using a Leica SP8 confocal microscope (Leica Microsystems). The remaining exosomes were stored at −80°C.

### 2.2 Animal model establishment

HBx transgenic male C57BL/6 mice (HBx transgenic mice), aged 3–6 weeks, were generously provided by Professor Zhou Haoxiong from the Third Affiliated Hospital of Sun Yat-sen University. Wild C57BL/6 male mice of corresponding age were procured from Beijing Weitong Lihua Experimental Animal Technology Co. Ltd. These mice were raised in a sterile-pathogen-free (SPF) animal facility under a 12–12 light/dark schedule with free access to food and water. After acclimatization for 1 week, normal C57BL/6 mice and HBx transgenic mice were randomly divided into different groups. The Con group consisted of 6 C57BL/6 mice injected with equal volume of 20 μL PBS. The HBx group included 6 HBx transgenic mice, of which 3 mice were injected with equal volume PBS through tail vein, and 3 mice were administered with equal volume normal saline orally. The remaining HBx transgenic mice were randomly divided into 5 different groups, including HBx + Exo (tail vein injection of 10 μg Exo), HBx + miR-223-3p KD Exo group (tail vein injection of 10 μg BMSC^miR-223−3p KD^-Exo), HBx + p-STAT3 inhibitor group (administration of 25 mg/kg Stattic), and HBx + Exo + OE-HDAC2 group (tail vein injection of Exo transfected with 10 μg overexpressing HDAC2 plasmid). The control HBx + Exo + NC group included Exo transfected with 10 μg OE-HDAC2 empty plasmid, injected into the tail vein. Treatment was administered every 3 days, and mice were euthanized after 3 weeks of continuous treatment. Kidney tissue samples were collected for analysis. Furthermore, a cohort of six HBx transgenic mice were randomly allocated to either the HBx + OE-HDAC2 group, in which HDAC2 overexpressing lentivirus was administered via tail vein injection at a dosage of 0.2 mL (1.00E+8 TU), or the HBx + NC group, which received an equivalent dose of negative lentivirus. After 8 weeks, the mice were subject to sacrifice for renal tissue sample collection. HDAC2 overexpressing lentivirus and negative lentivirus were synthesized by Genechem (Shanghai, China). The animal studies were conducted according to Laboratory animal - Requirements of environment and housing facilities (GB 14925–2001) and Regulations for The Administration of Affairs Concerning Experimental Animals, and approved by The Medical Ethics Committee of The Affiliated Hospital of Qingdao University.

### 2.3 Hematoxylin-eosin (HE) staining

Renal tissues were fixed in 4% paraformaldehyde for 24 h and embedded in paraffin, then cut into 4-μm-thick sections. The sections were stained using the HE staining kit (ab245880, Abcam) following the manufacturer’s instructions. Subsequently, the sections were observed under a light microscope (Nikon, Eclipse Ci-L, Japan).

### 2.4 Cell culture, transfection, and treatment

Human renal podocyte lines (HPCs, HTX2426) were sourced from otwo biotech (Shenzhen). HPCs were cultured in RPMI-1640 medium supplemented with 10% fetal bovine serum and 100 U/mL penicillin/streptomycin. HPCs were maintained in a constant incubator, containing 5% CO_2_ at 33°C. When the density of HPCs reached 70%, HPCs were transferred to a continuous temperature incubator containing 5% CO_2_ at 37°C for 10–14 days for differentiation.

After HPCs were differentiated and matured, they were transfected using the identical methodology as previously mentioned. The construction of HBx-induced HPCs model was established through the utilization of HBx overexpressing lentivirus. HPCs were transfected with 50 nM miR-223-3p mimic/inhibitor to induce the overexpression or knockdown of miR-223-3p. Additionally, HBx-induced HPCs were transfected with 2 μg histone deacetylase 2 (HDAC2) overexpression or empty plasmid. The HBx overexpressing lentivirus, inhibitor NC, miR-223-3p inhibitor, mimic NC, and miR-223-3p mimic were synthesized by Genechem (Shanghai, China), while the HDAC2 overexpression plasmid and empty plasmid were synthesized by HonorGene (Changsha, China). Furthermore, Stattic (HY-13818, MCE) served as a potent inhibitor of STAT3 phosphorylation. The cells were randomly divided into various groups, including the Con group (cultured under normal passage conditions without any additional treatment, serving as the experimental control), HBx group (HPCs transfected with lentivirus overexpressing HBx), HBx + p-STAT3 inhibitor group (HBx-induced HPCs treated with 1.0 μM Stattic), HBx + OE-HDAC2 group (HBx-induced HPCs transfected with a plasmid overexpressing HDAC2), HBx + NC group (HBx-induced HPCs transfected with an empty plasmid), HBx + OE-HDAC2+Exo group (HBx-induced HPCs transfected with a plasmid overexpressing HDAC2 co-cultured with BMSC-Exo), HBx + Exo group (HBx-induced HPCs co-cultured with BMSC-Exo), HBx + Exo + OE-HDAC2 group (HBx-induced HPCs co-cultured with BMSC-Exo transfected with a plasmid overexpressing HDAC2), and HBx + Exo + NC group (HBx-induced HPCs co-cultured with BMSC-Exo transfected with an empty plasmid).

### 2.5 Cell viability assay

Cell viability was evaluated utilizing the Cell Counting Kit-8 (CCK-8; Sigma, United States). The HPCs, treated according to their respective groups, were resuspended at a concentration of 1 × 10^5^ cells/mL, subsequently introduced into a 96-well plate. Following a 24, 48, 72, and 96 h period, 10 μL of CCK-8 was supplemented to each well, and subsequently incubated at 37°C for an additional 4 h. Absorbance values (OD) were measured at 450 nm using a microplate reader (Thermo Fisher, United States).

### 2.6 Reactive oxygen species (ROS) measurement

Intracellular ROS production was quantified using a reactive oxygen species assay kit (Meilunbio, Dalian, China). The treated HPCs were gathered through centrifugation at 1,000×g for 5 min, followed by treatment with 2′,7′-dichlorodihydrofluorescein diacetate (DCFH-DA) at a concentration of 10 μM or PBS at 37°C for 1 h. After being washed twice with PBS, the supernatant was discarded, and the cells were re-suspended in 500 μL of PBS. Finally, flow cytometry (Agilent, NovoCyte) was employed to analyze the levels of ROS.

### 2.7 Malondialdehyde (MDA) and Fe^2+^ determination

The expression levels of MDA and Fe^2+^ were measured using the Lipid Peroxidation (MDA) Assay kit (ab118970, Abcam) and Iron Assay kit (ab83366, Abcam), respectively, according to the manufacturer’s instructions.

### 2.8 RNA immunoprecipitation (RIP) assay

RIP assays were performed using the Magna RIP TM RNA-binding protein immunoprecipitation kit (Merck, 17-701) according to the manufacturer’s instructions. HPCs were lysed using RIP lysate, and 5 μg of HDAC2 antibody (ab32117, Abcam, 1:60) was pre-conjugated to protein A/G magnetic beads in immunoprecipitation buffer for 2 h, followed by incubation with 100 μL of cell lysates for 16 h at 4 C. IgG antibody (bs-0297P; Bioss) was used as a negative control. Co-precipitated RNA was extracted and evaluated by qRT-PCR. hsa-miR-223-3p forward primer TGT​GTG​GTG​TCA​GTT​TGT​CAA​AT, universal primer Qiagen (Cat No. 1046471), designed by Primer Premier, was synthesized by Beijing DynaScience Biology.

### 2.9 TEM

Three randomly selected samples from each group were subjected to electron microscope observation, and images were captured using a TEM (Hitachi, HT7700, Japan). Cells from each group were collected, fixed overnight with 2.5% glutaraldehyde, followed by fixation with 1% osmium tetroxide for 2 h. Subsequently, after undergoing gradient dehydration, the cells were embedded in epoxy resin. Similarly, kidney tissue specimens were also embedded in epoxy resin. The samples were then sectioned into ultra-thin slices (60–80 nm) and stained with lead citrate for 15 min. Five photographs were taken of each sample from different viewing angles, and the captured images were collected for subsequent analysis.

### 2.10 Immunofluorescence

The cells were fixed with 4% paraformaldehyde and then permeated with 0.1%Triton X-100 for 10 min. Kidney sections 4 μm thick were incubated with blocking buffer at room temperature for 30 min. Then anti-GPX4 antibody (1:200, Abclonal, A1933), HBx antibody (1:200, Abcam, ab2741) and nephrin antibody (1:100, boster biological technology, China), desmin antibody (1:100, boster biological technology, China) were added overnight. The secondary antibody was incubated at room temperature for 2 h. In addition, the samples were treated with Dapi dye for nuclear staining (358 nm). The fluorescence microscope uses the Leica SP8 confocal microscope.

### 2.11 Western blot analysis

Protein samples from cells and tissues were extracted and total protein concentration was determined using a BCA kit (Beyoncé, P0012, Shanghai, China). The total protein in the samples was separated by 6%–12% SDS-polyacrylamide gel electrophoresis and then transferred to 0.22 μm polyvinylidene difluoride membranes. Subsequently, the membranes were incubated with primary antibodies, followed by incubation with the corresponding secondary antibodies. The target bands were developed using the ECL chemiluminescence kit (Servicebio, Wuhan, China), and grayscale values were analyzed by ImageJ software. The following antibodies were used: HRP-labeled goat anti-rabbit (1:10,000, ZhongShan JinQiao, ZB-2301, Beijing); HRP-labeled goat anti-mouse (1:10,000, ZhongShan JinQiao, ZB-2305, Beijing); anti-GAPDH (1:50,000, proteintech, 60004-1-IG, United States); anti-HDAC2 (1:1,000, A19626, ABclonal); anti-STAT3 (1:1,000, A1192, ABclonal); anti-p-STAT3 (1:2,000, 9145T, CST); anti-GPX4 (1:1,000, ABclonal, A1933); anti-SLC7A11 (1:1,000, Proteintech, 26864-1-AP, United States); anti-ACSL4 (1:1,000, Abclonal, A6826); anti-CD9 (1:1,000, Abcam, ab92726); anti-TSG101 (1:1,000, Abcam, ab125011).

### 2.12 Quantitative real-time PCR

Total RNA was extracted using Trizol reagent (Convoy Century, CW0580S) and cDNA was synthesized using Evo M-MLV Reverse Transcription Reagent (Acres Bio, AG11706) according to the manufacturer’s instructions. cDNA was synthesized using SYBR Green Pro Taq HS Pre-mixed qPCR kit (Acres Bio, AG11701) on a fluorescent qPCR instrument (GAPDH was used as an internal reference). Data were analyzed using the 2^−ΔΔCT^ method. The primer sequences used in the study were detailed in Table 1. The primer sequence of miR-223-3p was provided by Ribobio (Guangzhou, China).

**TABLE 1:** Primer sequences.

Names	Sequences (5′–3′)
GPX4-F	GCTGGACGAGGGGAGGAG
GPX4-R	GGA​AAA​CTC​GTG​CAT​GGA​GC
ACSL4-F	ACT​GGC​CGA​CCT​AAG​GGA​G
ACSL4-R	GCC​AAA​GGC​AAG​TAG​CCA​ATA
SLC7A11-F	TCT​CCA​AAG​GAG​GTT​ACC​TGC
SLC7A11-R	AGA​CTC​CCC​TCA​GTA​AAG​TGA​C
HDAC2-F	TGG​CCT​TTC​TGA​GCT​GAT​TT
HDAC2-R	AGC​CAC​TGA​AAC​AAG​ACT​TCA
GAPDH-F	AGA​AGG​CTG​GGG​CTC​ATT​TG
GAPDH-R	AGG​GGC​CAT​CCA​CAG​TCT​TC
U6-F	CTCGCTTCGGCAGCACAT
U6-R	AAA​TAT​GGA​ACG​CTT​CAC​G

### 2.13 Statistical analysis

Each experiment was performed at least three times independently. The data were displayed as means ± standard deviation. GraphPad Prism version 8.0 software was used to analyze the data. Two-tailed unpaired Student's t tests were performed to evaluate statistical differences between two groups. Multiple-group comparisons were evaluated using one-way ANOVA analysis of variance followed by Tukey’s test. Statistical significance was set at *p* < 0.05.

## 3 Results

### 3.1 Inhibition of STAT3 phosphorylation attenuates HBx-induced ferroptosis in podocytes

**FIGURE 1:**
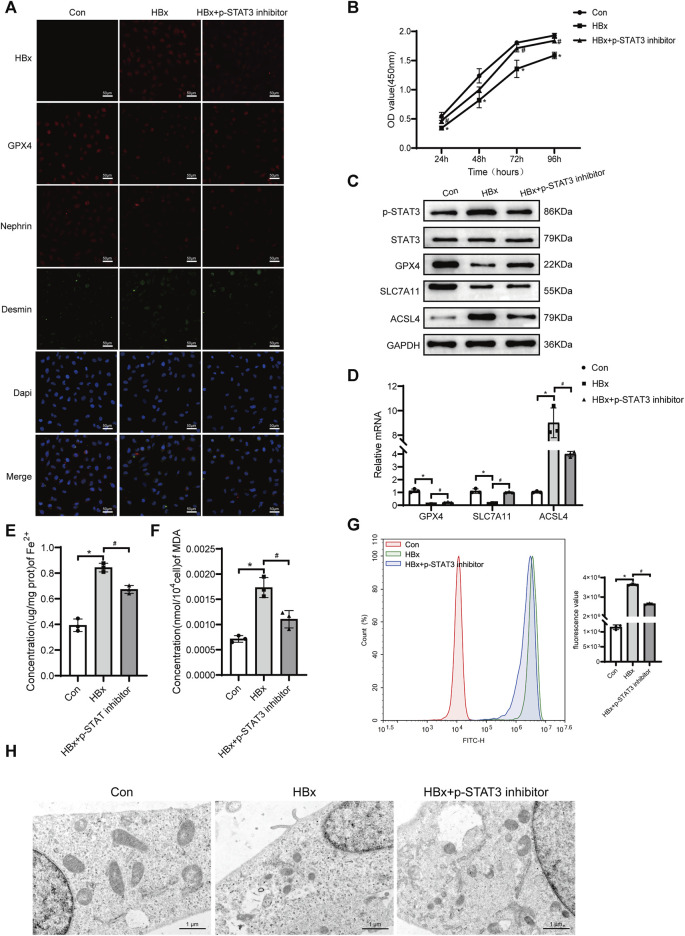
Inhibition of STAT3 phosphorylation enhanced the level of ferroptosis in HBx-induced HPCs. **(A)**Immunofluorescence assay of HBx, GPX4, nephrin, and desmin in HPCs (scale bar = 50 μm). **(B)**Cell viability was analyzed by CCK8 assay at 24, 48, 72, and 96 h. **(C)** Western blot analysis for the expression of p-STAT3, STAT3, GPX4, SLC7A11, and ACSL4. **(D)** RT-qPCR assay for the expression of GPX4, SLC7A11, and ACSL4. **(E, F)** Assay kits showing the level of Fe^2+^ and MDA. **(G)**The level of ROS was detected by flow cytometry. **(H)**The structure of mitochondria was observed by TEM (scale bar = 1 μm). Data are the means ± SEM (*n* = 3/group). *p < 0.05 vs. Con group; #*p* < 0.05 vs. HBx group.

Previous studies have shown that there is a correlation between STAT3 activation and the onset of ferroptosis (Luo et al., 2023). In this study, we investigated the potential role of p-STAT3 in regulating HBx-induced ferroptosis in podocytes. As shown in Figure 1A, transduction of lenti-HBx significantly enhanced the fluorescence intensity of HBx in HPCs, indicating that HBx was successfully transfected into HPCs. Immunofluorescence revealed that HBx caused podocyte damage, as evidenced by decreased expression of the podocyte marker nephrin and increased expression of the podocyte damage marker desmin (Figure 1A). At 24, 48, 72, and 96 h, HBx decreased the cell viability of HPCs (Figure 1B). Next, we investigated the impact of HBx on ferroptosis by examining the levels of GPX4, SLC7A11, and ACSL4, which are molecules associated with ferroptosis. In comparison to the controls, HBx decreased the levels of GPX4 and SLC7A11, while increasing the expression of ACSL4 at both the mRNA and protein levels (Figures 1C,D). Similar results were observed when immunofluorescence for GPX4 protein (Figure 1A). Additionally, we evaluated the levels of lipid peroxidation products MDA, Fe^2+^, and reactive oxygen species (ROS) to further confirm the impact of HBx on ferroptosis. The results presented in Figures 1E–G indicated that HBx significantly increased the levels of MDA, Fe^2+^, and ROS. Additionally, transmission electron microscopy (TEM) revealed a decrease in mitochondrial volume and an increase in mitochondrial membrane density, while the mitochondrial ridge displayed a decline (Figure 1H). Furthermore, it was observed that HBx elevated the protein level of p-STAT3 in HPCs (Figure 1C).

The podocytes transfected with HBx were treated with a p-STAT3 inhibitor. Figure 1C shows that the p-STAT3 inhibitor successfully reduced p-STAT3 protein levels. Notably, the inhibitor also decreased HBx expression in podocytes and improved the extent of podocyte injury, as evidenced by the expression of nephrin and desmin (Figure 1A). Furthermore, p-STAT3 inhibition mitigated the HBx-induced reduction in cell viability (Figure 1B). The effects of p-STAT3 inhibitors on ferroptosis-related markers were further investigated. p-STAT3 inhibition significantly reversed the effects of HBx on GPX4, SLC7A11, ACSL4, Fe^2+^, MDA and ROS levels (Figures 1C–G). TEM also showed that p-STAT3 inhibitors reversed HBx-induced ferroptosis in HPCs (Figure 1H). Collectively, these data suggested that p-STAT3 played a critical role in HBx-induced podocyte injury and ferroptosis.

### 3.2 HDAC2 promotes STAT3 phosphorylation through epigenetic modifications

**FIGURE 2:**
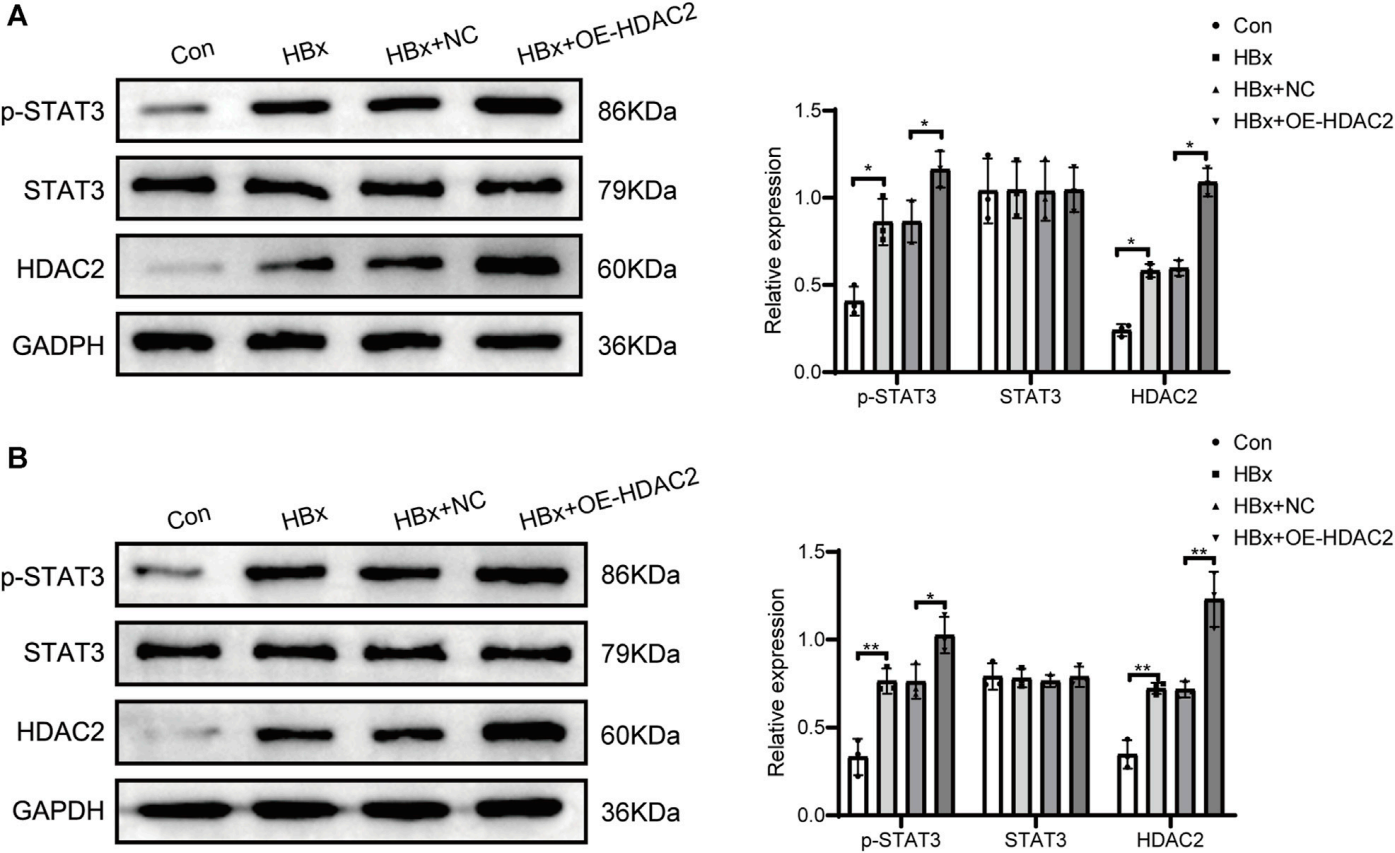
HDAC2 promoted STAT3 phosphorylation via epigenetic alterations. **(A)** Western blot analysis for the expression of HDAC2, STAT3, and p-STAT3 in HBx-induced HPCs and **(B)** HBx transgenic mice. Data are the means ± SEM (*n* = 3/group). *p < 0.05 vs. the respective NC group.

Previous studies have shown that HDAC2 plays an important role in the regulation of STAT3 activation (Pang et al., 2011). Subsequently, we investigated the impact of HDAC2 on STAT3 phosphorylation in the presence of HBx. HBx-induced HPCs were transfected with HDAC2 overexpression plasmid and empty plasmid, respectively. As shown in Figure 2A, overexpression of HDAC2 in podocytes enhanced the induction of HDAC2 protein expression by HBx. At the same time, overexpression of HDAC2 in podocytes enhanced HBx-mediated phosphorylation of STAT3 (Figure 2A). By injecting 0.2 mL (1.00E+8 TU) of HDAC2 overexpressing lentivirus and an equal amount of negative lentivirus into the tail vein of HBx transgenic mice, it was found that HBx transgenic mice showed significantly higher renal HDAC2 and p-STAT3 expression than control mice. Furthermore, the expression level of renal p-STAT3 was further increased after overexpression of HDAC2 (Figure 2B). These results suggested that HDAC2 has the ability to increase STAT3 phosphorylation through epigenetic modifications.

### 3.3 BMSC-Exo is taken up by podocytes

**FIGURE 3:**
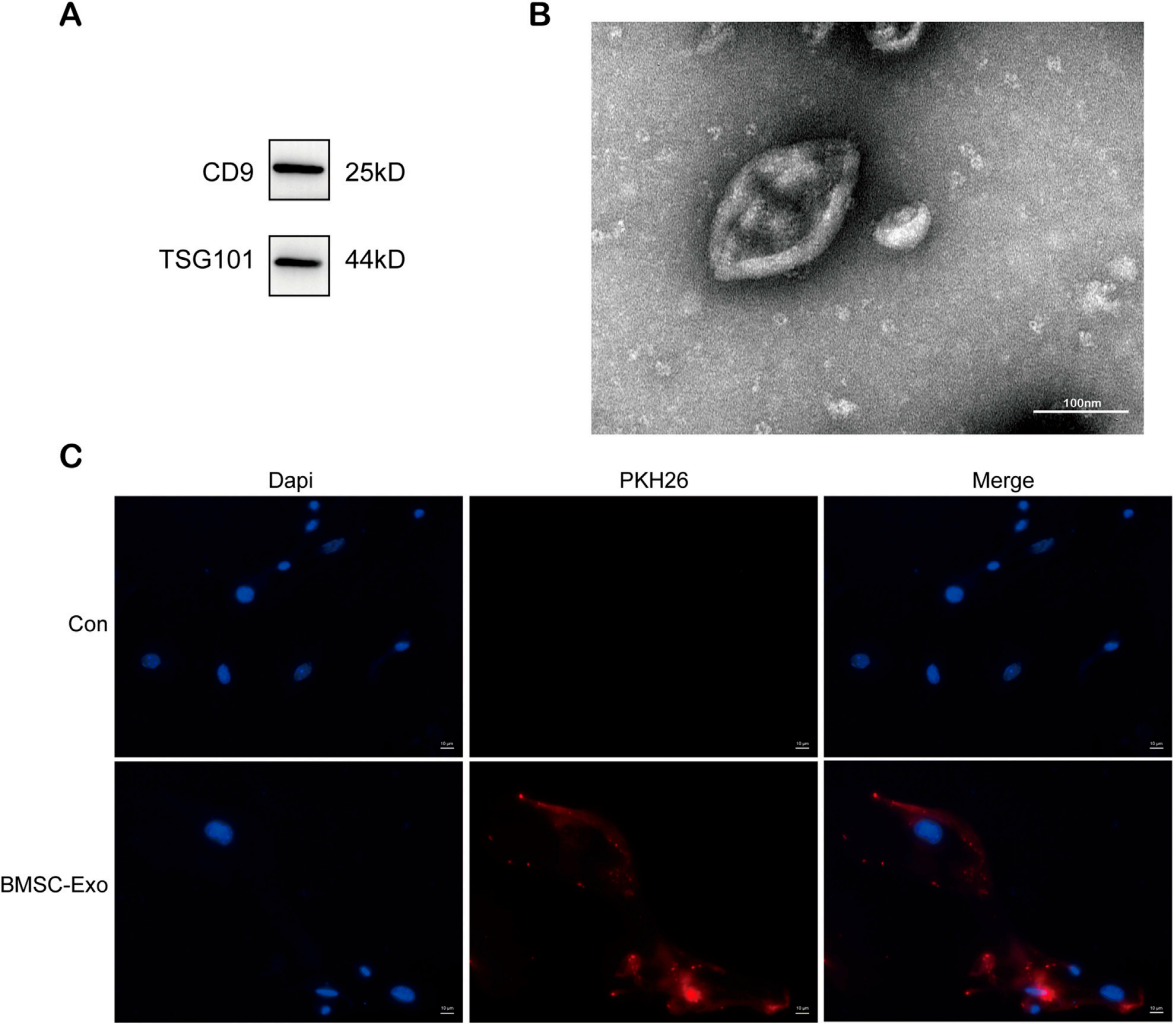
Characterization and uptake of BMSC-Exo. **(A)** Western blot analysis for the expression of CD9 and TSG101. **(B)** The morphology of BMSC-Exo was observed by TEM (scale bar = 100 nm) **(C)** PHK26 labeled BMSC-Exo was taken up by HPCs (scale bar = 10 μm).

In our previous study, exosomes from bone marrow mesenchymal stem cells containing miR-223-3p attenuated HBx-induced ferroptosis in HPCs (Yang et al., 2023). We isolated exosomes from serum-free medium in which BMSCs were cultured. As shown in Figure 3A, western blot revealed high expression of exosome marker proteins CD9 and TSG101. TEM revealed that the particles exhibited a round or elliptical shape (Figure 3B), with diameters ranging from 30 nm to 150 nm. Additionally, we used the lipophilic membrane dye PKH26 to label BMSC-Exo. When the red PKH26-labelled BMSC-Exo was incubated with HPCs, they were taken up by podocytes (Figure 3C).

### 3.4 BMSC-Exo regulates HDAC2 to attenuate podocyte injury and ferroptosis

**FIGURE 4:**
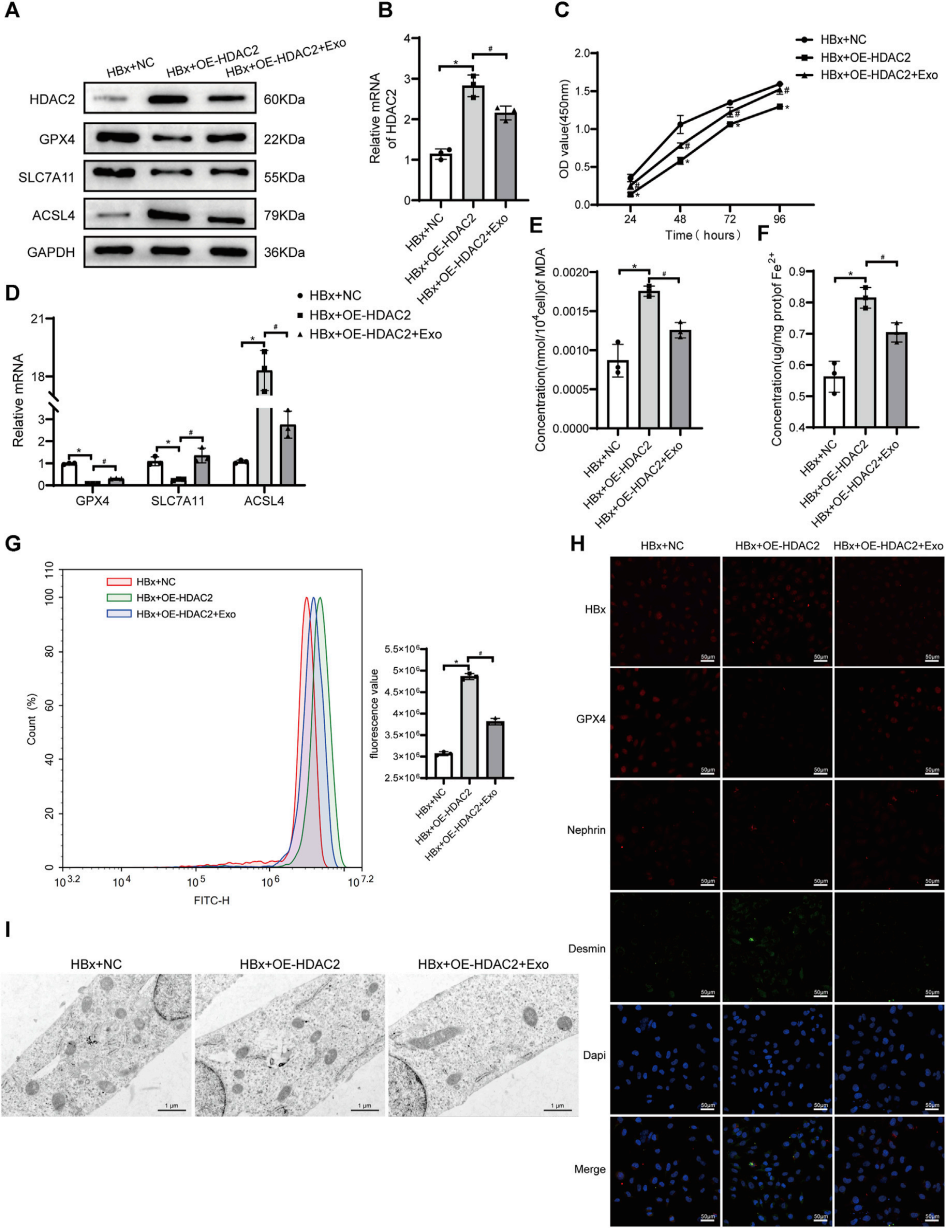
HBx-induced HPCs received the overexpression of HDAC2(OE-HDAC2) with or without Exo. **(A)** Western blot analysis for the expression of HDAC2, GPX4, SLC7A11, and ACSL4. **(B)** RT-PCR assay for the expression of HDAC2. **(C)** Cell viability was analyzed by CCK8 assay. **(D)** RT-PCR assay for the expression of GPX4, SLC7A11, and ACSL4. **(E, F)** Assay kits showing the level of MDA and Fe^2+^. **(G)**The level of ROS was detected by flow cytometry. **(H)** Immunofluorescence assay of HBx, GPX4, nephrin, and desmin in HPCs (scale bar = 50 μm). **(I)**The structure of mitochondria was observed by TEM (scale bar = 1 μm). Data are the means ± SEM (*n* = 3/group). *p < 0.05 vs. HBx + NC group; #*p* < 0.05 vs. HBx + OE-HDAC2 group.

We found that the level of p-STAT3 is closely associated with ferroptosis in podocytes, in which HDAC2 plays an important regulatory role. Previous studies have found that HDAC2 is involved in the epigenetic regulation of ferroptosis-related genes (Kong et al., 2023). We next explored the regulatory role of HDAC2 in ferroptosis in podocytes. We transfected HDAC2 overexpression plasmids or empty plasmids in HBx-induced HPCs. The expression of HDAC2 protein and mRNA was significantly increased in the HBx + OE-HDAC2 group compared with the HBx + NC group, indicating that the HDAC2 plasmid was successfully transfected into HPCs (Figures 4A,B). Cell viability was decreased after HDAC2 overexpression (Figure 4C). Meanwhile, indicators related to ferroptosis were measured, including Fe^2+^ content, oxidative stress-related markers, and the expression of ferroptosis-related proteins. The results showed that HDAC2 overexpression resulted in decreased expression of GPX4 and SLC7A11 and increased levels of Fe^2+^, MDA, ROS, and ACSL4 (Figures 4A,D–H). Similarly, HDAC2 overexpression led to a decrease in mitochondrial volume and an increase in mitochondrial membrane density in podocyte (Figure 4I). This implied that HDAC2 enhanced HBx-induced ferroptosis in podocytes. In addition, the degree of podocyte damage increased after HDAC2 overexpression, as manifested by a decrease in nephrin fluorescence intensity and an increase in desmin fluorescence intensity (Figure 4H).

Our previous study found that BMSC-Exo ameliorated podocyte ferroptosis (Yang et al., 2023). We speculated that BMSC-Exo might regulate podocyte ferroptosis by acting on HDAC2. After transfection of HDAC2 overexpression plasmid in HBx-induced HPCs, BMSC-Exo was administered. Compared to the HBx + OE-HDAC2 group, we observed a suppression of HDAC2 expression in podocytes co-cultured with BMSC-Exo. This prevented the decrease in cell viability caused by OE-HDAC2. (Figures 4A–C). BMSC-Exo consistently reversed the effects of HDAC2 overexpression on GPX4, SLC7A11, and ACSL4 (Figures 4A,D). Additionally, BMSC-Exo reduced lipid peroxidation levels in podocytes, as evidenced by the levels of MDA and ROS (Figures 4E,G). Moreover, BMSC-Exo alleviated intracellular iron accumulation in podocytes (Figure 4F). Furthermore, we observed the alleviation of mitochondrial damage and podocyte injury using TEM and immunofluorescence staining, respectively (Figures 4H,I).

### 3.5 HDAC2 is the target gene of miR-223-3p

**FIGURE 5:**
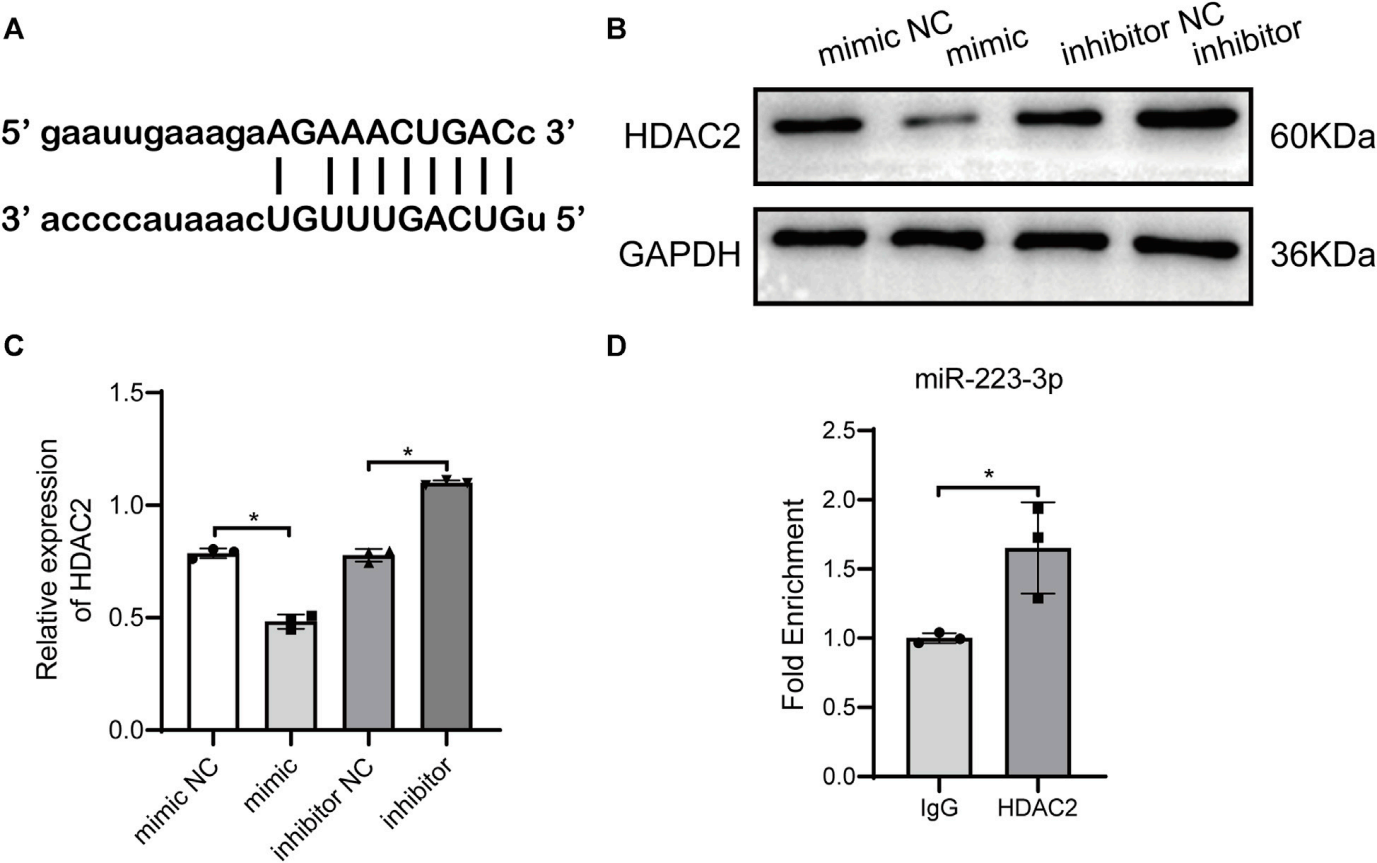
miR-223-3p directly targeted HDAC2. **(A)** Binding sites between miR-223-3p and the 3′-untranslated regions (UTRs) of NLRP3 were analyzed using bioinformatics methods. **(B, C)** Western blot analysis for HDAC2 expression with miR-223-3p mimic or inhibitor. **(D)** RNA binding protein immunoprecipitation assays detecting miR-223-3p and HDAC2 interaction in HPCs. Data are the means ± SEM (*n* = 3/group). *p < 0.05 vs. the respective NC group.

miR-223-3p-encapsulated BMSC-Exo reversed the promoting effect of overexpressed HDAC2 on HPCs ferroptosis and injury induced by HBx. Therefore, we assumed there may be an interaction between miR-223-3p in BMSC-Exo and HDAC2, which in turn acts on HBx-induced ferroptosis and damage of HPCs. Prediction of the gene target of miR-223-3p using the ENCORI database showed that miR-223-3p had binding sites to HDAC2 (Figure 5A). Applying miR-223-3p mimic and inhibitor, we detected the change of HDAC2 expression in HPCs, and the results showed that miR-223-3p could negatively regulate HDAC2 expression (Figures 5B,C). In addition, we detected the interaction between miR-223-3p and HDAC2 in HPCs using the RIP assay, showing that miR-223-3p was able to act directly on HDAC2 (Figure 5D). These results demonstrated that miR-223-3p regulated HDAC2 expression by directly targeting its mRNA 3′UTR sequence.

### 3.6 miR-223-3p in BMSC-Exo attenuates HBx-induced ferroptosis in HPCs by reducing STAT3 phosphorylation levels through HDAC2

**FIGURE 6:**
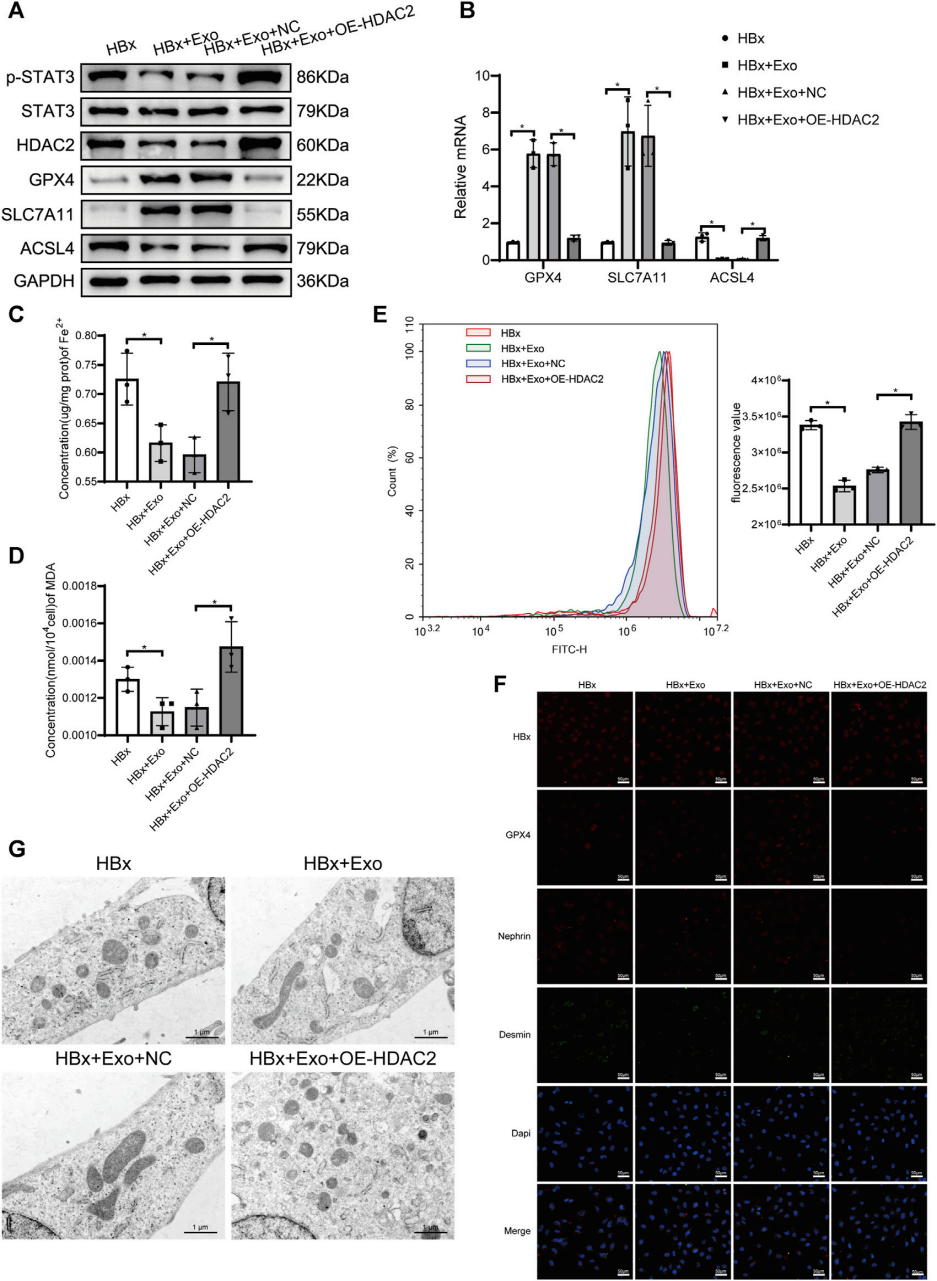
miR-223-3p in BMSC-Exo attenuated HBx-induced ferroptosis by reducing HDAC2-mediated STAT3 phosphorylation levels in HPCs. **(A)** Western blot analysis for the expression of p-STAT3, STAT3, HDAC2, GPX4, SLC7A11, and ACSL4. **(B)** RT-PCR assay for the expression of GPX4, SLC7A11, and ACSL4. **(C, D)** Assay kits showing the level of Fe^2+^ and MDA. **(E)** The level of ROS was detected by flow cytometry. **(F)** Immunofluorescence assay of HBx, GPX4, nephrin, and desmin in HPCs (scale bar = 50 μm). **(G)** The structure of mitochondria was observed by TEM (scale bar = 1 μm). Data are the means ± SEM (*n* = 3/group). *p < 0.05 vs. the respective NC group.

To explore whether HDAC2 plays a key role in miR-223-3p-encapsulated BMSC-Exo function, we overexpressed the level of HDAC2 in BMSC-Exo. Exosomes were extracted after transfection of HDAC2 overexpression plasmid into BMSC and added to HBx-induced HPCs. Compared with the HBx group, the HDAC2 expression level was decreased after the addition of BMSC-Exo, which might be caused by miR-223-3p targeting in BMSC-Exo (Figure 6A). Meanwhile, p-STAT3 expression level decreased with HDAC2 (Figure 6A). And the HDAC2 expression level was significantly increased after the addition of HDAC2 overexpressing BMSC-Exo, and the p-STAT3 expression level was subsequently increased (Figure 6A). By detecting the expression of ferroptosis-related genes in podocytes we found that BMSC-Exo decreased the expression of ACSL4 and enhanced the expression of GPX4 and SLC7A11 compared with the HBx group (Figures 6A,B,F). Meanwhile, BMSC-Exo attenuated HBx-induced iron accumulation, lipid peroxidation levels, and ameliorated the extent of mitochondrial damage (Figures 6C–E,G). The protective effect of miR-223-3p in BMSC-Exo was inhibited after overexpression of HDAC2 in BMSC-Exo (Figures 6A–G). Finally, by immunofluorescence we found that the addition of BMSC-Exo decreased the expression of desmin and elevated the expression of nephrin compared to the HBx group, and this alteration was reversed by the addition of BMSC-Exo overexpressing HDAC2 (Figure 6F). We therefore concluded that miR-223-3p in BMSC-Exo attenuated HBx-induced podocyte injury and ferroptosis by regulating HDAC2 expression and thereby altering STAT3 phosphorylation levels.

### 3.7 Inhibition of STAT3 phosphorylation attenuates ferroptosis in potocytes of HBx transgenic mice

**FIGURE 7:**
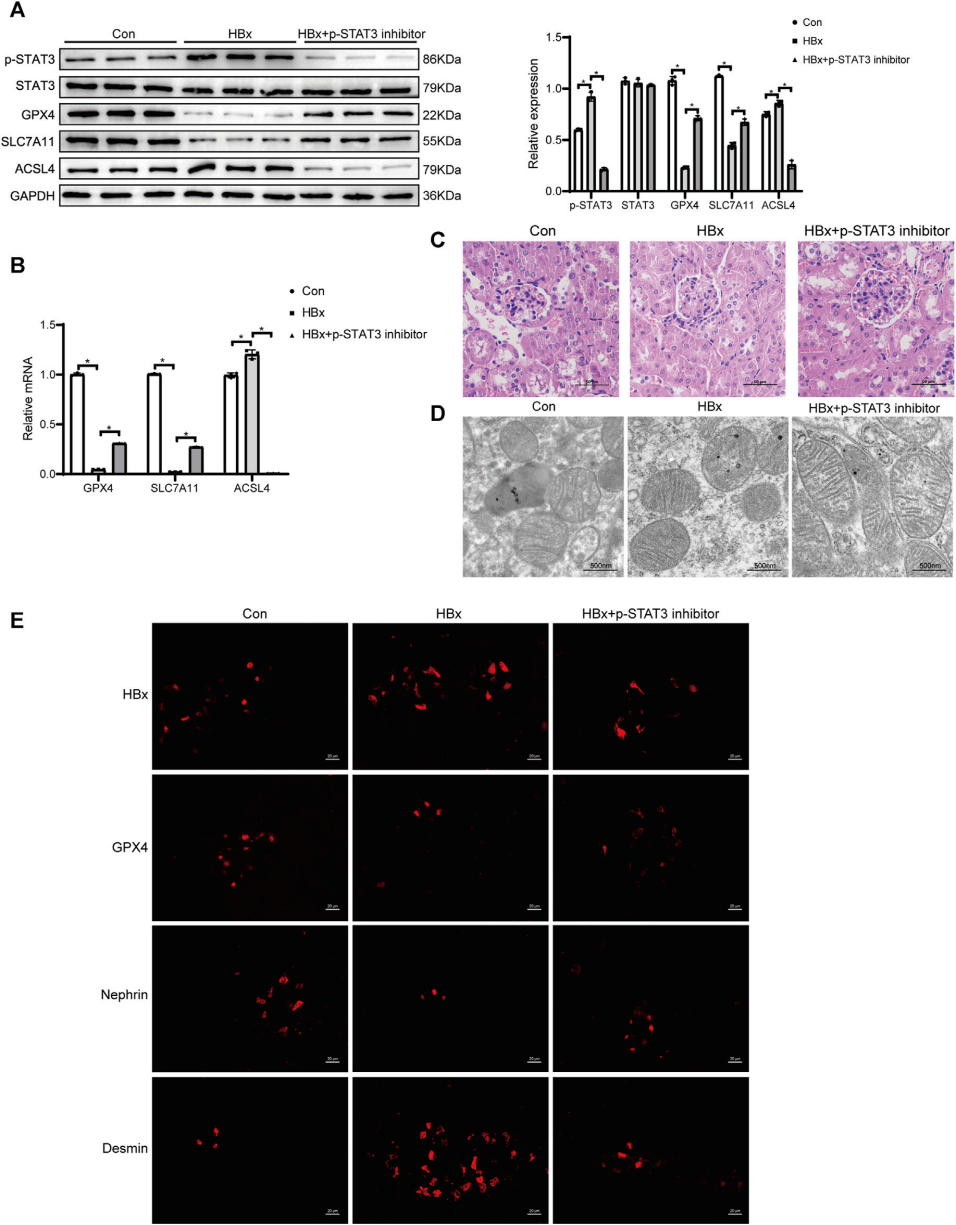
Inhibition of STAT3 phosphorylation enhanced the level of ferroptosis in potocytes of HBx transgenic mice. **(A)** Western blot analysis for the expression of p-STAT3, STAT3, GPX4, SLC7A11, and ACSL4. **(B)** RT-PCR assay for the expression of GPX4, SLC7A11, and ACSL4. **(C)** HE staining of kidney tissue of HBx transgenic mice (scale bar = 50 μm). **(D)** The structure of mitochondria was observed by TEM (scale bar = 500 nm). **(E)** Immunofluorescence assay of HBx, GPX4, nephrin, and desmin in kidney tissue of HBx transgenic mice (scale bar = 20 μm). Data are the means ± SEM (*n* = 3/group). *p < 0.05 vs. the respective NC group.

On the other hand, the relationship between STAT3 phosphorylation and ferroptosis in podocytes of HBx transgenic mice was verified by administering a STAT3 phosphorylation inhibitor via gavage. In HBx transgenic mice, renal HBx expression levels were significantly higher than those in the Con group mice (Figure 7E). HBx increased renal p-STAT3 expression, while STAT3 expression remained unchanged (Figure 7A). Additionally, renal GPX4 and SLC7A11 expression levels were lower, and ACSL4 expression levels were higher (Figures 7A, B). These results were further confirmed by immunofluorescence detection of GPX4 (Figure 7E). HE staining revealed Significant renal tubular oedema with glomerular stasis in the HBx group (Figure 7C). Electron microscopy showed reduced mitochondrial volume and increased bilayer membrane density in podocytes of the HBx group (Figure 7D). Additionally, there was an increase in the area of desmin fluorescence and a decrease in the area of nephrin fluorescence in podocytes (Figure 7E), and blood creatinine and urea nitrogen (BUN) levels were reduced (Supplementary Figure S1A,B). These results indicated that damage and ferroptosis occur in the renal and podocytes of HBx transgenic mice.

The mice were treated with 25 mg/kg of Stattic by gavage every 3 days for 3 consecutive weeks for sampling. In HBx transgenic mice treated with a STAT3 phosphorylation inhibitor, renal p-STAT3 expression was significantly reduced compared to HBx transgenic mice without treatment (Figure 7A). Additionally, HBx expression was also decreased (Figure 7E) and inhibition of STAT3 phosphorylation led to a reduction in renal HBx expression. However, the expression levels of renal GPX4, SLC7A11, and nephrin were elevated, while ACSL4 and desmin expression were downregulated (Figures 7A,B,E). Additionally, glomerular, tubular, and podocyte mitochondrial damage were reduced (Figures 7C,D). Supplementary Figure S1A,B illustrated the increase in blood creatinine and BUN levels. These findings suggested that inhibitors of STAT3 phosphorylation can partially reverse renal injury caused by HBx and podocyte ferroptosis.

### 3.8 miR-223-3p in BMSC-Exo alleviates ferroptosis in HBx transgenic mice by reducing STAT3 phosphorylation through HDAC2

**FIGURE 8:**
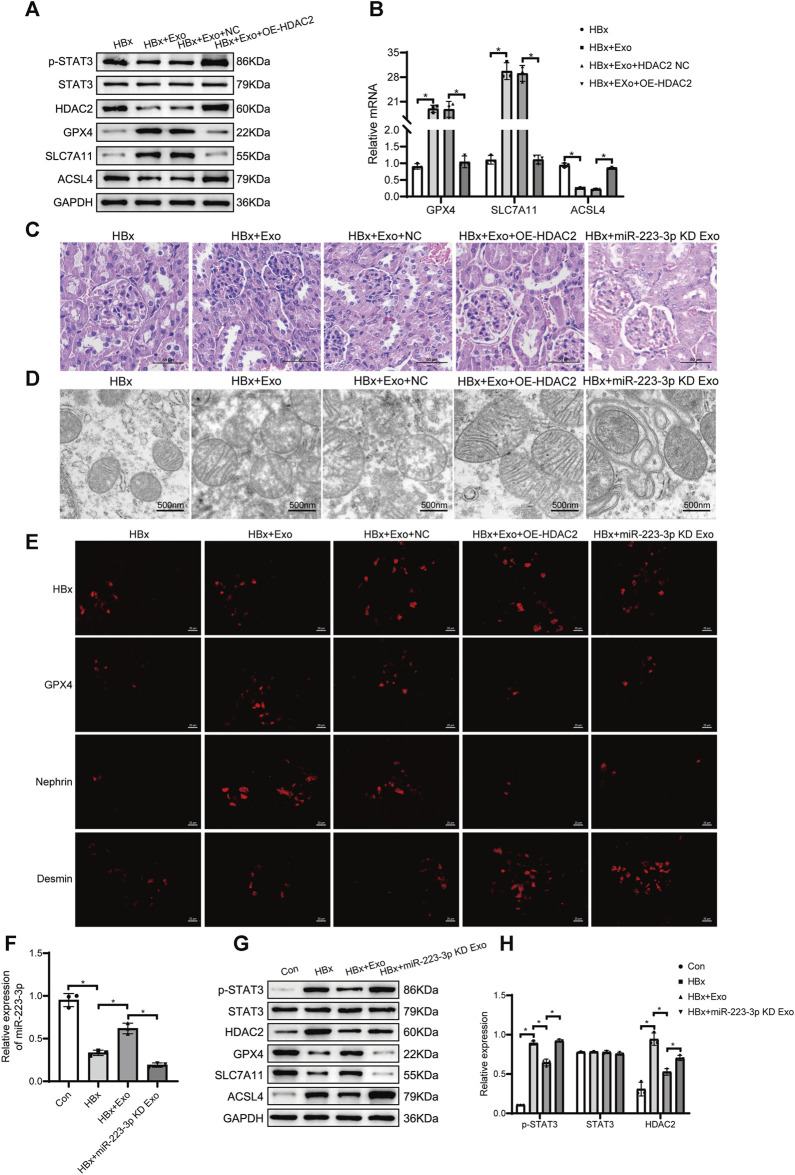
miR-223-3p in BMSC-Exo inhibited ferroptosis in HBx transgenic mice by reducing STAT3 phosphorylation through HDAC2. **(A)** Western blot analysis for the expression of p-STAT3, STAT3, HDAC2, GPX4, SLC7A11, and ACSL4. **(B)** RT-PCR assay for the expression of GPX4, SLC7A11, and ACSL4. **(C)**HE staining of kidney tissue of HBx transgenic mice (scale bar = 50 μm). **(D)** The structure of mitochondria was observed by TEM (scale bar = 500 nm). **(E)**Immunofluorescence assay of HBx, GPX4, nephrin, and desmin in kidney (scale bar = 20 μm). **(F)** RT-PCR assay for the expression of miR-223-3p. **(G)**Western blot analysis for the expression of p-STAT3, STAT3, HDAC2, GPX4, SLC7A11, and ACSL4. **(H)** RT-PCR assay for the expression of p-STAT3, STAT3, and HDAC2. Data are the means ± SEM (*n* = 3/group). *p < 0.05 vs. the respective NC group.

In this study, we proceeded to investigate the therapeutic impact of BMSC-Exo in HBx transgenic mice. The mice were administered with 10 μg of BMSC-Exo via the tail vein every 3 days for a duration of three consecutive weeks. A comparative analysis was conducted, which demonstrated a reduction in the expression levels of HDAC2 and p-STAT3 in the renal tissues of mice treated with BMSC-Exo. Additionally, the genes associated with ferroptosis, namely, ACSL4, exhibited downregulation, while GPX4 and SLC7A11 showed upregulation. These findings were in stark contrast to the untreated HBx transgenic mice (Figures 8A,B). In addition, BMSC-Exo treatment led to a reduction in the extent of glomerular tubular injury, an increase in podocyte mitochondrial volume, and a decrease in mitochondrial membrane density (Figures 8C,D). Immunofluorescence showed a significant increase in nephrin fluorescence intensity and a decrease in the extent of podocyte injury (Figure 8E). Blood creatinine and BUN levels were significantly reduced after BMSC-Exo treatment compared with HBx transgenic mice (Supplementary Figure S1A,B).

We further validated the role of HDAC2 in BMSC-Exo treatment of HBx transgenic mice. 10 μg of HDAC2 overexpressing BMSC-Exo was injected into the tail vein of HBx transgenic mice every 3 days, and the material was taken from the consecutive treatment for 3 weeks. Compared with tail vein injection of BMSC-Exo transfected with null expression plasmid, the treatment of BMSC-Exo after overexpression of HDAC2 was attenuated, as evidenced by the upregulation of renal ACSL4 and the downregulation of GPX4, SLC7A11 as well as an increased degree of mitochondrial damage in glomerular tubules and podocytes (Figures 8A–E). At the same time, renal p-STAT3 expression was significantly elevated (Figure 8A).

Interestingly, we found that miR-223-3p levels were significantly decreased in HBx transgenic mice and rebounded after BMSC-Exo treatment (Figure 8F), which validated our previous results in the *in vitro* model. Next, we transfected BMSC with miR-223-3p inhibitor, extracted BMSC^miR-223−3p KD^-Exo, and injected it into HBx transgenic mice via tail vein. We found that the renal HDAC2 and p-STAT3 expression levels were elevated in HBx transgenic mice with BMSC^miR-223−3p KD^-Exo compared with HBx transgenic mice treated with BMSC-Exo alone (Figures 8G,H), which implied that the knockout of miR-223-3p resulted in the loss of the miR-223-3p effect on the HDAC2 targeting, the expression levels of HDAC2 as well as the downstream p-STAT3 were elevated. In addition, renal ACSL4 was upregulated and GPX4 and SLC7A11 were downregulated in HBx transgenic mice with BMSC^miR-223−3p KD^-Exo, and the extent of glomerular as well as pedunculated cellular mitochondrial damage was increased (Figures 8C–E,G), blocking the therapeutic effect of BMSC-Exo in HBx transgenic mice. Taken together, our data indicated that miR-223-3p in BMSC-Exo had a regulatory effect on downstream HDAC2/p-STAT3 as well as a therapeutic effect on renal injury and podocyte ferroptosis in HBx transgenic mice.

## 4 Discussion

HBV infection is a major public health concern, with an estimated 316 million people worldwide living with chronic HBV infection in 2019 (GBD, 2019 Hepatitis B Collaborators, 2022). HBV-GN is the most common extrahepatic lesion caused by HBV infection and has become one of the main secondary kidney diseases in our country. HBx is a multifunctional regulatory protein encoded by the HBV genome and consists of 154 amino acids (Zhao et al., 2021). HBx can bind to HBV covalently closed circular DNA (cccDNA) to drive HBV virus infection through protein-protein interactions and participate in the process of HBV replication (You et al., 2022). In addition, HBx decreases podocyte adhesion and increased inflammatory cytokine secretion (He et al., 2017; Wang et al., 2016). HBx expression is negatively correlated with podocyte marker protein expression, and HBx has been found in the glomeruli of HBV-GN patients, suggesting that HBx plays an important role in the pathogenesis of HBV-GN (Guan et al., 2022).

Since the discovery of ferroptosis, there has been increasing evidence of the potential value of targeting ferroptosis as a therapeutic target for diseases (Lu et al., 2022). The regulatory pathways of ferroptosis can be mainly divided into three types, including glutathione/GPX4 pathway, iron metabolism regulation mechanism, and lipid peroxide accumulation. Previous studies provided direct genetic evidence that GPX4 knockout can lead to ferroptosis and found that the glutathione/GPX4 axis plays an important role in preventing renal lipid oxidation (Friedmann Angeli et al., 2014). Ferroptosis may be key in folate-induced AKI because ferrostatin 1 (Fer-1) improves renal function and reduces intracellular lipid peroxidation, tubule cell damage and death (Martin-Sanchez et al., 2017). In addition, ferroptosis aggravated diabetic nephropathy in diabetic mice, and treatment with Fer-1 alleviated kidney damage (Feng et al., 2021). Ferroptosis is involved in the occurrence and development of many renal diseases. However, the role of ferroptosis in HBV-GN remains unclear and needs further exploration. Previous studies have shown that HBx can increase the levels of mitochondrial ROS and lipid peroxidation in hepatocytes and induce oxidative damage (Lee et al., 2004). In this experiment, after transfection of HBx, the expression of marker proteins in podocytes decreased, which was consistent with previous studies (Wang et al., 2016; Guan et al., 2022). Meanwhile, the expression levels of genes related to ferroptosis in podocytes were changed, GPX4 and SLC7A11 were decreased, and the levels of ACSL4 and ROS were increased. The results indicated that ferroptosis was involved in the injury of HBx podocytes.

MSCs are pluripotent stem cells that are abundant, readily available, self-renewing, and present in a variety of tissues, including bone marrow, fat, and umbilical cord tissue, exhibiting anti-inflammatory and immunomodulatory properties (Murphy et al., 2013; Galipeau and Sensébé, 2018). Studies have shown that most MSC-mediated protective effects can be attributed to the function of released exosomes, and MSC-derived exosomes can provide effective therapeutic strategies for a variety of diseases and are novel and promising therapeutics based on regenerative medicine (Janockova et al., 2021). MSC-Exo is rich in lipids, proteins, miRNAs, and other forms of RNA, and is closely related to the resistance to oxidative stress and repair of tissue damage. MSC-Exo can inhibit ferroptosis of liver cells by promoting the function of SLC7A11, thereby mediating liver repair in mice with acute liver injury (Lin et al., 2022). In addition, exosomes derived from different stem cells are effective in preventing acute and chronic kidney disease *in vivo* and *in vitro* (Tetta et al., 2020). Many miRNAs have been found to be involved in the regulation of biological functions. miR-381-3p in BMSC-Exo could alleviate vascular calcification in chronic kidney disease by targeting NFAT5 (Liu et al., 2022). Similarly, BMSC-derived EVs inhibit renal fibrosis in mice by delivering miR-181d to neighboring cells (Wang et al., 2022). Our preceding investigations have demonstrated that the therapeutic effect of BMSCs on ameliorating ferroptosis in HBx-transfected podocytes is closely linked to the activation of miR-223-3p. Most studies on miR-223-3p have focused on acute kidney injury and chronic kidney disease, and few studies have investigated miR-223-3p for the treatment of HBV-GN. However, in previous studies, miR-223-3p demonstrated an amelioration of renal ischemia/reperfusion-induced injury (Yuan et al., 2017; Sun et al., 2022) and HBx induced downregulation of miR-223 levels (Yu et al., 2016), which was speculated that it might play a protective role in the development of HBV-GN. In this current study, we employed miR-223-3p-deficient BMSC-Exo to validate the renoprotective role of miR-223-3p in restraining ferroptotic damage in podocytes of HBx transgenic mice. Further investigations are warranted to shed light on the downstream targets of miR-223-3p and its underlying mechanisms in regulating ferroptosis.

STAT3 belongs to the STAT protein family, and STAT3 has important roles in fundamental biological processes, including proliferation, development, differentiation, inflammation, and apoptosis, and its activation is triggered by phosphorylation on key tyrosine residues. HBx protein was reported to be able to upregulate STAT3 and p-STAT3 levels by promoting the transcriptional activity of long non-coding RNAs in a nude mouse model of hepatocellular carcinoma (Chen et al., 2019). Clinical case studies have shown that p-STAT3 protein expression is significantly elevated in renal tissues of HBV-GN patients, and HBx activation of the JAK2/STAT3 signalling pathway upregulates the Bax/Bcl-2 ratio, which may be related to the pathogenic mechanism of HBV-induced nephritic tissue damage (He et al., 2013). In the present study, elevated p-STAT3 levels were detected in both HBx-transfected HPCs and the kidneys of transgenic mice, but there was no significant change in STAT3 expression levels. HBx expression levels in podocytes were similarly downregulated with the use of STAT3 phosphorylation inhibitors, suggesting that inhibition of STAT3 phosphorylation protects against HBx-induced podocyte injury. It was found that lipocalin 2 may promote the occurrence of ferroptosis in hypoxic-ischemic brain injury through activation of the NF-κB/STAT3 pathway (Luo et al., 2023). In addition, overexpression of STAT3 downregulated the protein levels of FTH1 and increased the toxicity of ferroptosis in cardiomyocytes induced by a high-fat diet (Zhu et al., 2023). In the present study, we found that inhibition of STAT3 phosphorylation levels in HBx transgenic mice increased renal GPX4 and SLC7A11 expression, decreased ACSL4 levels and ameliorated podocytes mitochondrial damage, suggesting that STAT3 phosphorylation is closely associated with HBx-induced ferroptosis in podocytes, which is consistent with previous results (Luo et al., 2023). However, thiostepton can inhibit STAT3 and GPX4 signaling to induce ferroptosis in pancreatic cancer cells, which is inconsistent with our findings (Zhang et al., 2022). STAT3 plays an important role in ferroptosis, but its effect may be related to the model constructed and the dose administered.

Epigenetic modifications, mainly including genomic DNA methylation and histone modifications, have been shown to play a crucial role in the regulation of renal inflammation (Weber et al., 2017). Histone modifications include acetylation, methylation, phosphorylation, and ubiquitination, and usually regulate gene transcription (Zhou et al., 2016). In cells, the level of histone acetylation is tightly controlled by histone acetyltransferase and histone deacetylase (HDAC). HDAC proteins are active in many pathological processes, including myocardial ischemia-reperfusion injury (Xie et al., 2014), retinal ischemic injury (Fan et al., 2013), and AKI (Cianciolo Cosentino et al., 2013). HBx is a protein involved in several pathogenic processes. It is able to alter host gene expression through epigenetic regulation, stimulation of HDAC and DNA methyltransferases (Tian et al., 2013). It was found that elevated HDAC2 expression was detected in HepG2 (human hepatoma cell line) after HBx overexpression (Yoo et al., 2008). In addition, HDAC activity was positively correlated with viral replication in the serum of HBV patients, and HDAC2 levels decreased significantly after antiviral therapy (Zhang et al., 2019). In the present experiment we likewise found significantly elevated HDAC2 expression levels in HBx overexpressing podocytes, implying that HDAC2 is involved in HBx-induced ferroptosis in podocytes.

Previous studies have found that HDAC1/2 promotes arterial endothelial cell surface vascular cell adhesion molecule-1 expression and atherosclerosis through inhibition of STAT3 acetylation-dependent GATA6 promoter methylation (Hu et al., 2021). In addition, HDAC1 and HDAC2 downregulation was associated with dephosphorylation and hyperacetylation of STAT3 (Pang et al., 2011). Thus, it was inferred that STAT3 may be a direct target of HDAC2. In the present experiments, by overexpressing HDAC2 in HBx-transfected HPCs as well as in HBx transgenic mice, we found elevated levels of p-STAT3 expression, which was accompanied by a deepening of ferroptosis in cells, indicating that HDAC2 can regulate p-STAT3 expression and promote ferroptosis in HBx transgenic mice. We then used the ENCORI database to predict the gene targets of miR-223-3p, showing that miR-223-3p has binding sites to HDAC2, and used RIP experiments to verify that miR-223-3p can directly bind to HDAC2, inhibit the expression level of HDAC2, which in turn regulates the phosphorylation level of STAT3 and attenuates the ferroptosis of podocytes levels as well as the extent of kidney injury.

This study aimed to investigate the effect of miR-223-3p in BMSC-Exo on ferroptosis in HBx transgenic mice. Our results showed that miR-223-3p in BMSC-Exo reduced iron overload, ROS accumulation, MDA overexpression, and GPX4 depletion in podocytes. Additionally, miR-223-3p inhibited transcription by binding to the HDAC2 promoter region and regulated ferroptosis in podocytes through the STAT3 pathway. These findings reveal a new target for the treatment of HBV-GN and provide new evidence for MSC therapy. However, several limitations in this study must be addressed. Firstly, there is a dearth of investigations addressing the expression levels of HDAC2 and p-STAT3 in extensive cohorts. Secondly, BMSC-Exo may contain other components that can treat podocyte ferroptosis and kidney injury, which require further identification and exploration. Lastly, the direct targeting of p-STAT3 by HDAC2 needs further verification. In this study, HBx transgenic mice were used to simulate the *in vivo* environment of HBV-GN. Many previous studies have used HBx transgenic mice to mimic the process of HBV infection and related liver disease development, and there is a lack of research on the regulation and role of HBx in HBV-GN for kidney injury. A recent study demonstrated that DNMT1 was upregulated in HBx overexpressing podocytes, which was able to lead to hypermethylation of the VDR promoter and subsequent activation of the NF-κB signalling pathway to promote epithelial mesenchymal transition and release of inflammatory mediators, providing new evidence that HBx induces renal injury in HBV-GN (Guan et al., 2022). In our previous studies, HBx was found to be involved in the onset and development of HBV-GN by activating the NLRP3 inflammatory vesicle signalling pathway through inhibition of miR-223 expression or inducing overexpression of PLA2R on the membrane of the podocyte, leading to podocyte pyroptosis (Yu et al., 2022; Feng et al., 2023). In the present study, *in vitro* experiments demonstrated that HBx induced podocyte injury as well as ferroptosis and reduced podocyte activity, whereas HBx transgenic mouse podocytes similarly underwent renal injury as well as podocyte ferroptosis, further validating the feasibility of the use of HBx transgenic mice for exploring the pathogenesis of HBV-GN. However, fewer experiments have been conducted to study HBV-GN in the HBx transgenic mouse model, and the HBx transgenic mice are not yet able to completely mimic the entire pathological process of kidney injury by HBV, and more experiments are needed to verify this. Future studies will further explore the therapeutic effect of miR-223-3p in exosomes on HBV-GN and the relationship between ferroptosis and other cell types in HBX-induced podocyte injury.

## Data Availability

The datasets presented in this study can be found in online repositories. The names of the repository/repositories and accession number(s) can be found in the article/Supplementary Material.
