# Supplementary material for: Exosomal miR-223-3p from bone marrow mesenchymal stem cells targets HDAC2 to downregulate STAT3 phosphorylation to alleviate HBx-induced ferroptosis in podocytes
Source: Front Pharmacol. 2024 Feb 20;15:1327149. doi: 10.3389/fphar.2024.1327149 (PMC10912342; doi:10.3389/fphar.2024.1327149)
Supplement: Supplementary file 1 [file DataSheet1.docx]

**Renal function**

Blood was taken from the eyes of mice after anaesthesia. Blood creatinine and urea nitrogen levels were measured by creatinine assay kit and urea assay kit (rayto, Shenzhen).

**Supplementary figure 1**


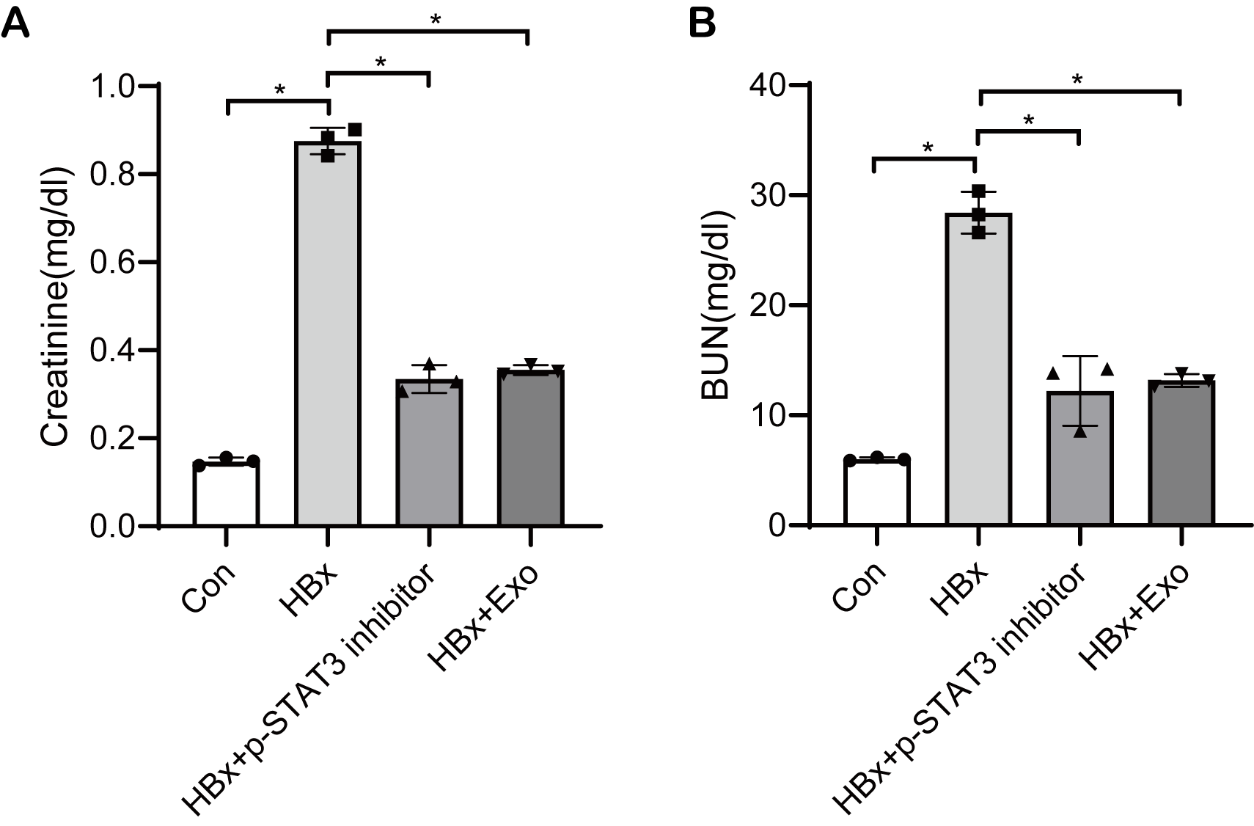


Figure.S1 Detection of renal function in mice of different treatment groups. Detection of (A) blood creatinine and (B) BUN in the Con group of mice, the HBx transgenic mice group, the HBx transgenic mice treated with BMSC-Exo group, and the HBx transgenic mice treated with p-STAT3 inhibitor group. Data were represented as mean ± SD from three independent experiments. **P* < 0.05.
